# Regulation of Cell Signaling Pathways by Berberine in Different Cancers: Searching for Missing Pieces of an Incomplete Jig-Saw Puzzle for an Effective Cancer Therapy

**DOI:** 10.3390/cancers11040478

**Published:** 2019-04-04

**Authors:** Ammad Ahmad Farooqi, Muhammad Zahid Qureshi, Sumbul Khalid, Rukset Attar, Chiara Martinelli, Uteuliyev Yerzhan Sabitaliyevich, Sadykov Bolat Nurmurzayevich, Simona Taverna, Palmiro Poltronieri, Baojun Xu

**Affiliations:** 1 Department of Molecular Oncology, Institute of Biomedical and Genetic Engineering (IBGE), Islamabad 44000, Pakistan; ammadfarooqi@rlmclahore.com; 2 Department of Chemistry, GCU, 54000 Lahore, Pakistan; qureshienv@yahoo.com; 3 Department of Bioinformatics and Biotechnology, International Islamic University, Islamabad 44000, Pakistan; sumbul.khalid@iiu.edu.pk; 4 Department of Obstetrics and Gynecology, Yeditepe University Hospital, 34755 Istanbul, Turkey; ruksetattar@hotmail.com; 5 Istituto Italiano di Tecnologia, Smart Bio-Interfaces, Pontedera, 56025 Pisa, Italy; chiara.martinelli@iit.it; 6 Department of Postgraduate Education and Research, Kazakhstan Medical University KSPH, Almaty 050004, Kazakhstan; e.uteuliyev@ksph.kz; 7 Department of General Surgery, Kazakhstan Medical University KSPH, Almaty 050004, Kazakhstan; gkb5gkb5@mail.ru; 8 Department of Biomedical Science, Institute of Biomedicine and Molecular Immunology “A. Monroy”, National Research Council, 90146 Palermo, Italy; simonataverna.unipa@gmail.com; 9 Department of Agrifood, National Research Council Italy Institute of Sciences of Food Productions (CNR-ISPA) Via Lecce-Monteroni km 7, 73100 Lecce, Italy; palmiro.poltronieri@ispa.cnr.it; 10 Food Science and Technology Program, Division of Science and Technology, Beijing Normal University-Hong Kong Baptist University United International College, Zhuhai 519087, China

**Keywords:** berberine, signaling pathways, oncogenic cascades, TRAIL, microRNAs, cancer therapy

## Abstract

There has been a renewed interest in the identification of natural products having premium pharmacological properties and minimum off-target effects. In accordance with this approach, natural product research has experienced an exponential growth in the past two decades and has yielded a stream of preclinical and clinical insights which have deeply improved our knowledge related to the multifaceted nature of cancer and strategies to therapeutically target deregulated signaling pathways in different cancers. In this review, we have set the spotlight on the scientifically proven ability of berberine to effectively target a myriad of deregulated pathways.

## 1. Introduction

Berberine, a natural alkaloid compound, is found in several medicinal plants. Typically, berberine is commercially produced from a Chinese medicinal plant *Coptis chinensis*. Berberine has captivated a substantial proportion of appreciation because of its remarkable pharmacological properties. Recent advancements in high-throughput techniques have helped us to demystify various hierarchically organized signaling complexes which play an instrumental role in cancer development and progression. Basic reviews related to the ability of berberine to improve worsening conditions in different diseases have previously been published, so our aim is not to summarize pharmacological importance of berberine in different diseases, but we have restricted our discussion specifically to berberine mediated targeting of signaling cascades in different cancers. In this review, we present current views related to how berberine effectively targets different deregulated oncogenic cascades and highlight key practical and conceptual questions that will be helpful to shape the next dimension of investigation into the ability of berberine to efficiently target different signaling cascades. We will start our overview with one of the most widely investigated cancer killing molecules: TNF-related apoptosis-inducing ligand (TRAIL).

## 2. Berberine Mediated Restoration of TRAIL-Mediated Apoptosis

There has always been a quest to identify the molecules having significant cancer killing activity and minimal off-target effects. In accordance with this view, the discovery of TRAIL revolutionized the field of molecular oncology [1,2]. However, initial claims were partially challenged by contemporary researchers because TRAIL was ineffective against different cancers. In-depth studies revealed that TRAIL transduced the signals intracellularly through death receptors (DR4, DR5) [3,4]. However, loss of cell surface appearance of death receptors was a frequently noted mechanism in TRAIL-resistance cancers. Moreover, imbalance of pro- and anti-apoptotic proteins was also reported in TRAIL resistant cancers. The advent of high-throughput technologies has helped us to uncover the highly orchestrated nature of TRAIL mediated signaling, which is initialized through extrinsic and intrinsic pathways. In this section, we will summarize recent developments and discuss unresolved and outstanding research questions.

Berberine has been shown to potently induce AMP-activated protein kinase (AMPK) in cancer cells [5]. Expectedly, berberine mediated apoptosis inducing effects were severely impaired in AMPKα-dominant negative (DN) expressing or AMPKα knockdown cancer cells. Knockdown of DR5 significantly abrogated TRAIL-berberine-induced apoptosis [5]. TRAIL and berberine combinatorially enhanced p38-MAPK phosphorylation [6]. p38-MAPK inhibition enhanced apoptosis in EGFR (epidermal growth factor receptor)-overexpressing MDA-MB-468 TNBC cells [6].

TRAIL and berberine significantly activated caspase-3 and cleavage of PARP in TRAIL-resistant MDA-MB-468 BCa cells [7]. In a murine 4T1 BCa model, berberine potentiated the efficaciousness of the anti-DR5 antibody and effectively blocked tumor growth and lung metastases [7]. Mcl-1 and c-FLIP have been shown to negatively regulate TRAIL-mediated apoptosis. Berberine dose-dependently induced degradation of Mcl-1 and c-FLIP [8]. However, treatment with a proteasome inhibitor MG132 interfered with berberine-mediated downregulation of Mcl-1 and c-FLIP [8].

While great efforts over the past few years have advanced our understanding of the berberine mediated regulation of the TRAIL-mediated pathway, much of this work has focused on preliminary information about the ability of berberine to improve TRAIL-induced killing activity. There are still many questions which need detailed research, for example, how berberine mechanistically regulates expression of death receptors in different cancers. Does it inhibit receptor degradation, or does it interfere with epigenetic silencing to restore expression of death receptors? How does berberine improve formation of death inducing signaling complex (DISC) while simultaneously targeting negative regulators which inhibit DISC formation? In the following section, we will discuss how berberine modulates the WNT/β-catenin pathway to inhibit cancer.

## 3. Regulation of WNT Pathway by Berberine

**Figure 1:**
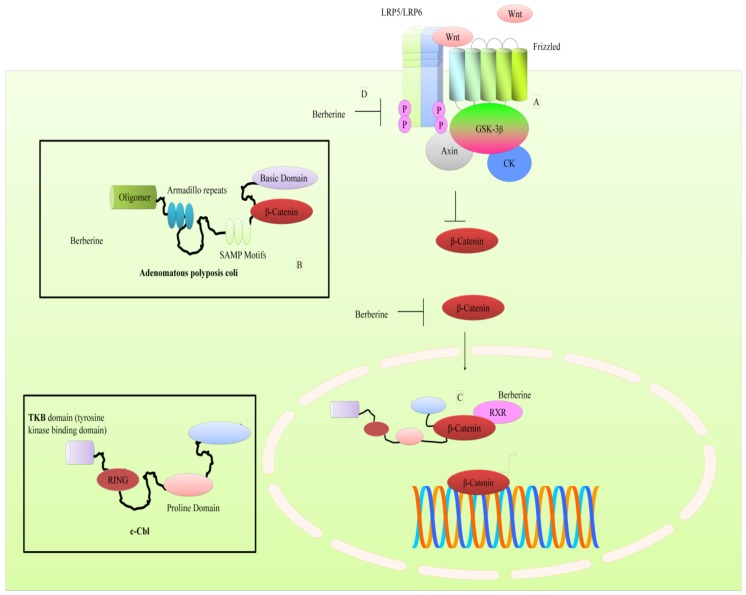
(**A**) WNT/ β-catenin mediated intracellular signaling. β-catenin moved into the nucleus to stimulate expression of target genes. (**B**) Berberine promoted interaction of β-catenin and APC to enhance degradation of β-catenin. (**C**) Berberine also promoted interaction of β-catenin with c-Cbl in nucleus that also induced degradation of β-catenin. (**D**) Berberine inhibited phosphorylation of LRP5/6 and GSK-3β. Abbreviations: c-Cbl (CASITAS B-lineage lymphoma protooncogene), LRP5/6 (Low density lipoprotein receptor-related protein), CK (Casein Kinase), GSK-3β (Glycogen synthase kinase), RXR (Retinoid X-receptor), WNT (Wingless/Integrase), SAMP (Ser-Ala-Met-Pro motif), RING (Really interesting new gene).

Levels of cytoplasmic β-catenin are controlled by a multi-proteins destruction complex which induces β-catenin phosphorylation, which is required for β-catenin ubiquitination and its subsequent degradation by proteasomes [9]. Berberine efficiently inhibited nuclear accumulation of β-catenin. Co-immunoprecipitation studies revealed that berberine increased the interaction between APC (adenomatous polyposis coli) and β-catenin [9] (shown in Figure 1). These findings shed light on the ability of berberine to stimulate the expression of APC and negatively regulate β-catenin by increasing physical interaction between these proteins.

There is evidence of mechanistic regulation of the WNT pathway by berberine in hepatocellular carcinoma cell line (SMMC-7721). However, these findings should be tested in other cancers. In a report, researchers demonstrated that berberine worked synergistically with HMQ1611, a taspine derivative and suppressed the phosphorylation of LRP5/6 and GSK3β [10]. Berberine and HMQ1611 combinatorially downregulated WNT5A, Frizzled8, CK1 (casein kinase 1) and APC. Overall this study uncovered distinct steps of β-catenin phosphorylation and degradation by berberine and HMQ1611 [10]. Importantly, berberine was unable to significantly inhibit tumor growth individually in mice xenografted with SMMC-7721 cells.

Berberine decreased levels of WNT5A and cytoplasmic β-catenin in both SGC7901 and AGS cells [11]. Treatment with berberine and galangin decreased β-catenin and WNT3A in esophageal carcinoma cells [12]. Berberine concentration-dependently downregulated mRNA expression of β-catenin in colon cancer cells [13]. RXRα (retinoid X receptor α) agonists have been shown to promote RXRα binding to β-catenin to induce ubiquitination and degradation of β-catenin [14]. Berberine dose-dependently enhanced physical association of RXRα with β-catenin. Berberine induced nuclear translocation of E3 ubiquitin ligase c-Cbl which modulated degradation of β-catenin [14] (shown in Figure 1). Overall, these findings clearly suggested that berberine enhanced degradation of β-catenin by promoting its interaction with c-Cbl.

It seems clear that berberine has potential to regulate the WNT pathway in different cancers, but it needs to be tested tactically in xenografted mice. Future studies must converge on identification of the modes opted by berberine to inhibit the WNT pathway in different cancers. Does it interfere with importin and exportin proteins to inhibit nuclear accumulation? Are there further previously unexplored ubiquitin ligases which can target β-catenin? Can berberine effectively target LRP5/6 and Frizzled receptors in other cancers to also efficiently inhibit cancer proliferation? In the upcoming section we will highlight how berberine regulates the Janus kinases-signal transducer and activator of transcription proteins (JAK-STAT) pathway in different cancers.

## 4. Targeting of JAK-STAT Pathway

Kinases of the Janus kinase (JAK) family and transcriptional factors of the STAT (signal transducer and activator of transcription) family form a highly dynamic and orchestrated membrane-to-nucleus signaling module that has been extensively investigated, and an overwhelmingly increasing list of scientific reports have provided evidence of natural products mediated targeting of the JAK-STAT pathway in different cancers. More importantly and excitingly, coupling of massively parallel DNA sequencing with chromatin-immunoprecipitation has enabled researchers to capture thousands of STAT-binding sites.

**Figure 2:**
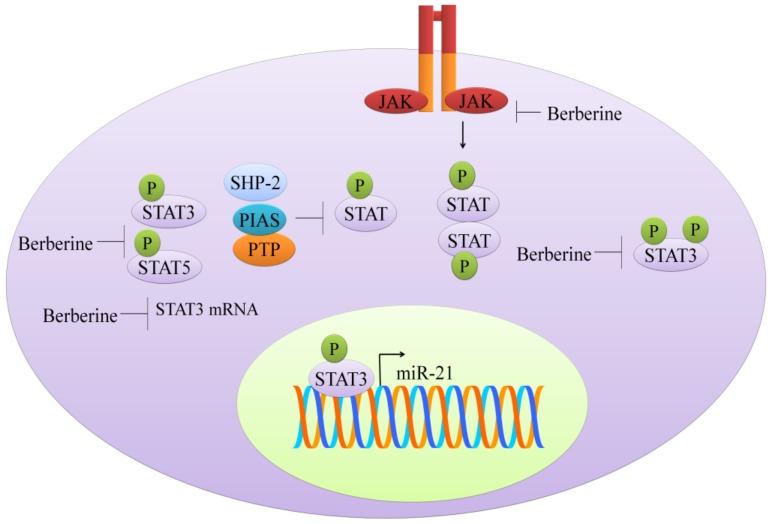
JAK-STAT signaling. Berberine has been shown to inhibit phosphorylation of STAT3 and STAT5. Berberine markedly reduced mRNA levels of STAT3. Functionally active STAT3 moved into the nucleus and stimulated expression of miR-21. Abbreviations: STAT (signal transducer and activator of transcription), JAK (Janus kinase), PIAS (Protein inhibitors of activated STATs), PTP (Protein-tyrosine phosphatase), SHP-2 (Src homology phosphatase-2).

Berberine reduced protein levels of STAT3 and inhibited the phosphorylation at 705th tyrosine and 727th serine in cholangiocarcinoma cell lines [15] (shown in Figure 2). Berberine exerted inhibitory effects on constitutive and IL-6-triggered activation of STAT3 in NPC (nasopharyngeal carcinoma) cells [16]. TAFs (tumor-associated fibroblasts) secreted IL-6 and the conditioned media harvested from the fibroblasts induced STAT3 activation in NPC cells. Activation of STAT3 by conditioned media of TAFs was blocked by berberine [16].

Berberine significantly decreased the phosphorylated levels of JAK2 and STAT3 in colorectal cancer cells [17]. Interestingly, p-JAK2 and p-STAT3 were found to be remarkably enhanced in COX2 (cyclooxygenase-2) overexpressing colorectal cancer cells. COX2 overexpression induced activation of JAK-STAT signaling further upregulated matrix metalloproteinases (MMP)-2 and MMP-9 in colorectal cancer cells. However, berberine effectively interrupted COX2/JAK/STAT signaling [17].

Colonization of *Fusobacterium nucleatum* in the intestine may contribute to colorectal cancer (18). Levels of p-STAT3 and p-STAT5 were found to be enhanced after inoculation of *F. nucleatum* in C57BL/6-APC^Min/+^ mice and wild-type C57BL/6 mice. Moreover, *F. nucleatum*-induced increases in quantities of p-STAT3 and p-STAT5 were found to be considerably reduced in mice treated with berberine [18] (shown in Figure 2).

Doxorubicin, a widely used chemotherapeutic drug has been shown to induce activation of STAT3 in lung cancer cells [19]. However, berberine markedly inhibited doxorubicin-triggered STAT3 activation. Besides, berberine promoted degradation of STAT3 by enhancing ubiquitination [19]. Berberine also enhanced killing effects of 5-fluorouracil by STAT3 inactivation and repressing the expression of survivin in gastric cancer cells [20].

## 5. Targeting of the mTOR Pathway: Could Berberine Modify Extracellular Vesicle-Composition?

The mammalian target of rapamycin (mTOR), a serine/threonine kinase of the PI3K (phosphoinositide 3-kinases)-related kinase family, is conserved on an evolutionary scale that coordinates different cellular processes. mTOR forms two structurally and functionally active complexes: mTOR complex 1 (mTORC1) and 2 (mTORC2). These two multi-component complexes are involved in physiological and pathological functions, such as macromolecules synthesis, homeostasis maintenance, cytoskeleton remodeling, angiogenesis, survival, response to stress and autophagy [21]. Considering the key role of mTOR in cell proliferation and differentiation, its deregulation contributes to cancer onset and progression [22].

The cellular metabolism mediated by mTOR is involved in the connections between cancer cells and tumor microenvironment during cancer advance and drug resistance acquisition, indicating the potential benefits of PI3K-Akt (protein kinase B)-mTOR pathway blockage. This inhibition contributes to reduce proliferation, migration, and survival of cancer cells, and increase tumor immunosurveillance through down-regulation of immunosuppression and anti-tumor immune stimulation [23]. mTORC1 is a downstream component in several pathways frequently altered in cancer, including the PI3K/Akt and MAPK (mitogen-activated protein kinases) pathways, that induces mTORC1 hyperactivation in many human cancers. Besides, mTORC2 signaling has a key role in tumors for its role in Akt activation, that induces tumor growth mechanisms such as glucose metabolism and apoptosis inhibition [24]. Recent reports show that mTOR is involved in lipid metabolism [25]; the critical step of this signaling cascade is the activation of proteins by phosphorylation at different sites. An important upstream target of mTOR is ERK (extracellular signal-regulated kinases), that regulates mTOR negatively and in turn enhances autophagy. ERK, a protein of MAPK cascade, is a central integrator of extracellular signals which are transduced by single cytokines or hormones or activated by cellular mechanical stresses that influence lipid metabolism [26,27].

The mTOR inhibitors, called “rapalogs,” used as anti-cancer drugs belonged to a class of rapamycin derivatives. The first rapalog, approved for advanced renal carcinoma management was temsirolimus, followed by everolimus. These rapalogs did not show significant results in clinical practice as compared to the results obtained in pre-clinical studies. The rapalogs and catalytic mTOR inhibitors were useful in immunosuppression in a small number of cancers [28,29].

Several natural compounds such as berberine, resveratrol, curcumin, quercetin and others can modulate the mTOR pathway [30,31,32]. Recent studies have revealed that berberine has anti-tumor effects, through inhibition of the mTOR-signaling pathway. Berberine, anisoquinoline alkaloid isolated from *Berberis vulgaris* L, has anti-diarrheic, anti-inflammation, and anti-microbial activities [33]. Nowadays, several studies have shown that berberine is effective against glioma, colorectal, lung, prostate and ovarian cancer [26].

Berberine can modulate different pathways, such as cellular glucose metabolism and the HIF-1α (hypoxia-inducible factor 1α)-mTOR axis. In this context, Wang et al. [26] indicated that berberine modulated the metabolism of glioblastoma multiforme cells, induced autophagy and reduced glucose metabolism. These changes reduced tumor growth and invasiveness, induced apoptosis, by AMPK/mTOR/ULK1 (Unc-51 like autophagy activating kinase) pathway inhibition. Berberine reduced cancer progression in vivo, which clearly indicated the potential clinical benefits of alkaloids extract from plants in cancer therapy [26].

Mao et al. provided evidence that berberine played a central role in regulation of cellular glucose metabolism in colon cancer cells [34]. They studied the effects of berberine in colon cancer cell lines and findings revealed that berberine inhibited glucose uptake and reduced the transcription of genes, such as *GLUT1* (glucose transporter 1), *LDHA* (lactate dehydrogenase A) and *HK2* (hexokinase 2), involved in glucose metabolism of colon cancer cells. This mechanism is mediated by HIF-1α protein synthesis inhibition through mTOR pathway suppression. The molecular study indicated that HIF-1α protein expression, a well-known transcription factor critical for dysregulated cancer cell glucose metabolism, was considerably inhibited in berberine-treated colon cancer cell lines [34]. It was reported that berberine activated AMPK that in turn inhibited mTOR, in in vitro studies and in mouse models of colon carcinogenesis in early stages of tumorigenesis. Berberine also interfered with the NF-κB (nuclear factor kappa-light-chain-enhancer of activated B cells) pathway and effectively inhibited colon cancer progression [33].

Berberine may also induce autophagy in human liver carcinoma cell lines, through activation of Beclin-1 and inhibition of mTOR signaling by suppressing the activity of Akt and up-regulating P38 MAPK signaling [35,36]. The role of berberine in mTOR pathway modulation has been also demonstrated in hematological malignancies. Ma and collaborators showed the synergism of TPD7 and berberine in leukemia Jurkat cell growth inhibition through ephrin-B2 signaling modulation [37]. There are direct pieces of evidence which shed light on synergistic antitumor activities of rapamycin and berberine treatment in hepato-carcinoma cell lines. There was a marked decrease in phosphorylated p70S6 kinase 1 protein levels, a downstream effector of mTOR in cells combinatorially treated with rapamycin and berberine as compared to cells treated with rapamycin or berberine alone [38]. It was also demonstrated that berberine and cinnamaldehyde reduced the susceptibility of mice to lung carcinogenesis induced by urethane, and reversed the urethane-induced AMPK, mTOR, AQP-1 (aquaporin 1) and NF-κB expression patterns [39]. Overall these reports advocated the role of berberine as a new compound for cancer therapy.

Recent findings indicate that extracellular vesicles (EVs) play a key role in different steps of cancer progression, transporting oncogenic proteins and nucleic acids [40,41,42]. EVs are named in different ways based on their origin, diameter and mechanism of release. The two population of EVs better characterized are exosomes and micro-vesicles [43,44]. Hypoxia induces wide changes in the tumor microenvironment, and several reports show EVs central role in this mechanism [45]. Besides HIF-1, other pathways such as PI3K/Akt/mTOR are induced in tumor cells under hypoxia. It was demonstrated that hypoxia promoted prostate cancer progression and hypoxis-induced-exosomes remodeled the cancer microenvironment [46]. Moreover, exosomes released by mesenchymal stem cells (MSC) dose-dependently reduced VEGF (vascular endothelial growth factor) expression and secretion mainly through interfering with the mTOR/HIF-1α axis in breast cancer cells. MSC-derived exosomes, enriched in miR-100, were taken up by breast cancer cells [47]. microRNA-100 efficiently downregulated VEGF in breast cancer cells.

This evidence suggests the possible role of berberine in modifying EV-composition, as has already been demonstrated for other natural compounds such as curcumin. It is exciting to note that exosomes released by curcumin-treated CML cells contained considerably higher levels of miR-21. Consequently, these miR-21 loaded exosomes were taken up by HUVECs (human umbilical vein endothelial cells) and it was mechanistically shown that miR-21 directly targeted MARCKS (myristoylated alanine-rich C-kinase substrate) and inhibited angiogenic phenotypes [48]. Curcumin also induced selective packaging of miR-21 in exosomes and played a central role in reshaping post-transcriptional network in recipient cells [49]. Furthermore, in CML cells, curcumin modulates other molecular pathways thus altering the metabolism of glucose that in myeloproliferative disease is a consequence of non-hypoxic activation of HIF-1α [50]. It was demonstrated that curcumin promoted miR-22 mediated targeting of importin 7 that resulted in a significant reduction in nuclear accumulation of HIF-1α [51].

It will be interesting to see if berberine demonstrated potent activity to promote release of exosomes loaded with tumor suppressor microRNAs and proteins.

## 6. Regulation of Epigenetic Modulators by Berberine

Histone marks are motives enabling the recruitment of chromatin complexes that activate or repress transcription. Histone modifications such as acetylation and methylation at specific positions are signals recognized by these complexes. Berberine was shown to upregulate some histone deacetylases (HDAC) of class II, such as sirtuin SIRT1 (sirtuin 1), producing an antiatherogenic effect, and suppression of foam cell formation in THP-1-derived macrophages treated with oxidized low-density lipoprotein [52]. RNA silencing of SIRT1 or AMPK blocked the berberine action.

Rel proteins have emerged as complex modulators of carcinogenesis and we still have to explore their functionalities in malignancies and response to cancer therapeutics [53]. Set9 (lysine methyltransferase) induced methylation of the RelA/p65 subunit, which inhibited nuclear accumulation of NF-κB and repressed transcriptional upregulation of miR-21. Berberine dose-dependently induced generation of ROS, arrested cancer cells in G(2)/M phase and induced apoptosis in U266 cells [53]. Overall, the findings clearly suggested that berberine promoted Set9 mediated methylation of p65 to limit shuttling of NF-κB into the nucleus. Berberine mediated inhibition of translocation of NF-κB into the nucleus resulted in inhibition of miR-21 and B-cell lymphoma 2 (Bcl-2).

The protective effects of berberine against metabolic syndrome might rely on increasing mitochondrial SIRT3 activity and stimulating glycolysis, independent of AMPK activation [54,55].

The growth arrest and DNA damage-inducible protein GADD45α (growth arrest and DNA damage 45α) is a DNA demethylation regulator recruited by TCF21 antisense RNA inducing demethylation (TARID) lncRNA to enable transcription of the TCF21 gene coding for tumor-suppressor gene transcription factor 21 [56]. As detailed in the next paragraphs, administration of *Coptidis rhyzoma* aqueous extract, resulted in a higher expression of miR-23a, and in up-regulation of GADD45a, a chromatin relaxer, decreasing DNA methylation through recruitment of the 5-hydroxymethylcytosine (5hmC) hyperproducing enzyme TET, and thymine DNA glycosidase (TDG) [57,58,59].

Berberine induced a decrease in activity of two DNA methylases, DNMT1 (DNA (cytosine-5)-methyltransferase 1) and DNMT3, that, through DNA hypomethylation, induced an increase of p53 [60]; p53 activation was observed following the increase in miR-23a, through repression of Nek6, in HCC cells in response to Rhizoma Coptis aqueous extract [60].

Various long noncoding RNA (lncRNAs), through their tertiary structure, work as scaffolds to recruit protein complexes such as chromatin modifiers, polycomb repressive complex (PRC), and transcriptional regulators to euchromatin regions: many lncRNAs have been associated with pharmacological drugs as well as to cisplatin treatment [61]. In various studies, berberine has been able to induce or repress some lncRNA [62].

## 7. Regulation of microRNAs by Berberine

Many bioactive compounds have been proposed for their regulatory effects on non-coding RNAs, either long ncRNAs (lncRNA), or small RNAs such as microRNAs [63,64,65]. These miRNAs have added new layers of complexity in the context of post-transcriptional regulation, controlling the availability of mRNAs, and selection of the mRNAs that they recognize as targets by complementary seed sequences to initialize the degradation process of mRNAs. miRNAs exert a regulatory role in the post-translational process.

OncomiRs are oncogenic due to their ability to support cell proliferation, apoptosis inhibition, cell stemness, while tumor suppressor miRNAs are involved in nodes or networks leading to differentiation, cell cycle inhibition and growth arrest and apoptosis.

There are feedback loops and feedforward loops, sustaining the oncogenic activity of ncRNAs: for instance, a feedback loop has been described in lymphoma between miR-17-92, MYC, the protein kinase Chk2 and hu antigen R (HUR) [66]. Transcription factors can induce the expression of protein-coding genes as well as of miRNAs, that can target the mRNAs of the induced genes, in a feedforward loop. In lymphoma cells, miR-17-92 regulates MYC mRNA levels through the inhibition of Chk2, causing the depression of RNA-binding protein HUR, and its binding to MYC mRNA, preventing MYC translation. Thus, berberine suppresses the growth of multiple myelomas, either by down-regulation of three oncogenic miRNA clusters and other mRNAs, or by involvement of p53 and MAP kinases [67].

Several researchers pointed out to a correlation between berberine treatment and expression of non-coding RNAs, either lncRNAs or microRNAs. In cancer studies, treatment of multiple myeloma cells with berberine, significantly suppressed three oncogenic miRNA clusters, miR-17-92, miR-106-25, and miR-99a-125b. Berberine mediated downregulation of miR-99a-125b was found to be correlated with the regulation of p53, MAP Kinases and ErbB oncogene, leading to cell cycle arrest in the G2-phase and to apoptosis [63].

**Figure 3:**
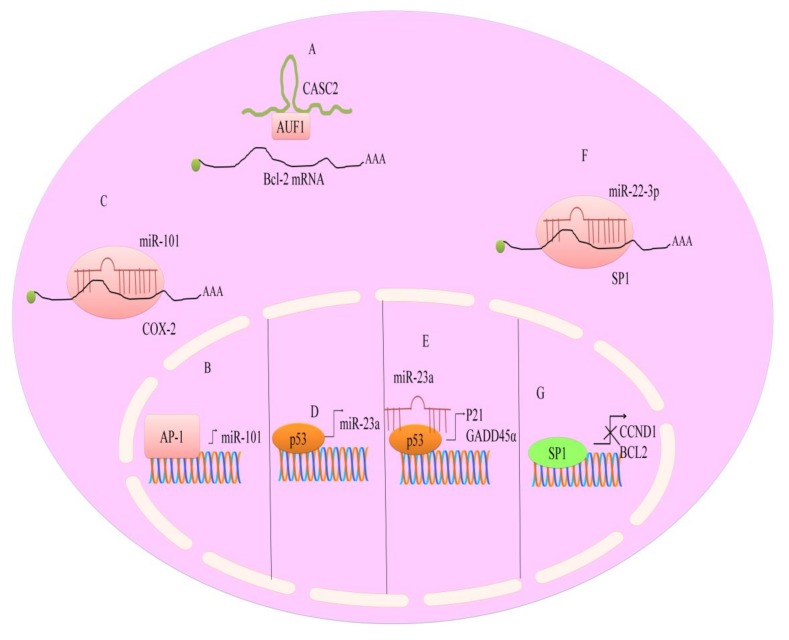
Regulation of non-coding RNAs by berberine. (**A**) CASC2 interacted with AUF1 and prevented its binding to AU rich sequences present within mRNA of Bcl-2. (**B**,**C**) AP-1 transcriptionally upregulated miR-101. miR-101 directly targeted COX-2. (**D**–**G**) P53 induced transcriptional upregulation of miR-23a. Additionally, miR-23a worked synchronously and stimulated the expression of GADD45a and p21. miR-22-3p directly targeted SP1. SP1 mediated upregulation of CCND1 and BCL2. Abbreviations: AUF1 (AU-rich element RNA-binding protein-1), BCL2 (B-cell CLL/lymphoma-2), *GADD45α* (Growth arrest- and DNA damage-inducible gene), CCND1 (Cyclin D1), CASC2 (long non-coding RNA), SP1 (specificity protein-1), AP-1 (Activator protein-1).

In colon cancers, berberine was effective in downregulating miR-429, with increase in its target, SOX-2; berberine up-regulated miR-296-5p and effectively interfered with the Pin1–β-catenin–cyclin D1 signal transduction cascade. Berberine inhibited the growth of HepG2 cells and induced the upregulation of miR-22-3p. miR-22-3p directly targeted SP1 and suppressed expression of its target genes, BCL2 (B-cell lymphoma 2) and CCND1 (cyclin D1) [68] (shown in Figure 3).

It was shown that berberine suppresses interleukin 6 (IL6), a factor required for cell growth in multiple myeloma cells (U266), through negative regulation of the signal transducer and activator of transcription 3 (STAT3), and this induces inhibition of miR-21 expression [69]. STAT3 regulates miR-21 expression through binding to STAT3 binding sites in the promoter (shown in Figure 2). Additionally, in ovarian cancer cells (SKOV3), berberine sensitized to cisplatin treatment through inhibition of miR-21, and subsequent activation of PDCD4, a tumor suppressor.

It is not straightforward to determine the role of miRNAs in tumor promotion or suppression, that depends on the context-specific, cell-type specific dual role of certain miRNAs [66]. This depends on the varieties of targets of miRNAs. There are miRNAs, such as miR-25 and miR-125b, that act as tumor suppressors in some tumor types and as oncogenes in others. In particular, in stem cells, miRNAs that in other cell types are regulated and respond to the bioactive supplements, may not decrease in levels, for their role in cell stemness. Berberine, in the form of a Rhizoma Coptis aqueous extract, resulted not effective in decreasing miR-21 levels, while it supported a higher expression of miR-23a, by up-regulating p21/GADD45a tumor suppressor gene (shown in Figure 3), causing HCC cells to arrest the growth in G2/M phase [19,70]. Furthermore, miR-23a was shown to repress Nek6 and to regulate p53 transcriptional activity.

Autophagy influences glucose and lipid metabolism in adipocytes. Berberine was shown to decrease miR-30a and miR-376b, preventing basal autophagy in 3T3-L1 adipocytes. MiR-30 interacts with the 3′-untranslated region of Beclin 1 (BECN1), thus the reduction in miR-30a levels increased BECN1 to form BECN1 complexes that induce autophagy (beclin homolog in yeast, Atg6, is known as autophagy promoting factor) [71]. This leads to reduced fat deposits and an increase in brown fat tissue.

Berberine was shown to exert an anti-apoptotic role in development of preimplantation embryos in vitro, by maintaining high levels of miR-21. Berberine up-regulated Bcl-2, in 2- and 4-cell embryos and blastocysts, and down-regulated caspase-3 and PTEN.

When the pre-miRNA is processed by the RISC complex, the guide strand is considered the mature, active miRNA, while the complementary passenger strand (termed miRNA*) is thought to be devoid of function. However, some miRNA star has been related to a function, some have a feedback role to regulate the RISC processing, and some have been shown to have anti-oncogene activity. This seems the case for miR-21*, or miR21-3p, affecting cancer cell growth [72]. In HepG2 hepatocellular carcinoma cells, berberine increased the levels of miR-21-3p, with a role in tumor growth inhibition and induction of apoptosis. In hepatic cancers, miR-21* antitumor activity relies on inhibition of MAT2A and MAT2B methionine adenosyltransferases mRNAs, with consequent increased levels of S-adenosyl-methionine (SAM). Methionine adenosyltransferase (MAT) played a central role in growth of hepatoma cells. miR-21-3p had previously been shown to directly target MAT2A and MAT2B in HepG2 cells. Berberine induced apoptosis in HepG2 cells mainly through miR-21-3p mediated targeting of MAT2A and MAT2B [72].

Ovarian cancer cells resistant to cisplatin, such as A2780 and A2780/DDP lines, were incubated with berberine combined with cisplatin, showing a significantly lower survival rate [73]. This effect was found to be related to inhibition of miR-93 expression, that translated in re-expression of PTEN tumor suppressor and recovery of AKT signaling [73]. Bcl-w has been shown to be targeted by miRNAs. In gastric cancers [60], berberine upregulated miR-203 and restored cisplatin-sensitivity in gastric cancer cells [74].

In liver cancers, berberine inhibited cell proliferation and viability in HepG2, Hep3B, and SNU-182 lines. Berberine treatment increased the expression of tumor suppressor such as Kruppel-like factor 6 (KLF6), activating transcription factor 3 (ATF3) and p21, a cell cycle inhibitor, and down-regulated the oncogene E2F transcription factor 1 (E2F1). A possible mechanism of the upregulation of protein coding genes may be hypothesized through downregulation of the respective miRNAs.

Berberine supplementation led to the miR29-b suppression, increasing insulin-like growth factor-binding protein (IGFBP1) expression in the liver; miR29-b suppression caused an increase in AMPK activity and a reduction of lipid storage in diabetic and obese patients [60]. Activation of AKT positively affected glucose uptake, reducing the glucose levels in blood.

LncRNAs are deregulated after berberine treatment in hepatocellular cancer, similarly to the effects seen after curcumin treatment on lncRNAs and on epigenetic changes seen in hepatocellular cancer [75]. A lncRNA, ANRIL, expressed at increased levels in type 2 diabetic (T2D) patients, causing an increase of CREB (cAMP response element-binding protein) expression, may be affected by berberine. Berberine down-regulated miR-122 and this caused a decrease in SREBP-1 levels in palmitic acid-treated HepG2 cells. Berberine was able to reduce the levels of glucose in T2D patients and in nonalcoholic fatty liver disease, by down-regulating lncRNA052686 and miR-122 [61,62,71,76,77]. The hyperlipidemic effect of berberine was linked to the inhibition of C/EBPα and PPARγ2 expression through inhibition of phosphorylated CREB binding to C/EBPβ promoter. MRAK052686, a lncRNA downregulated in diabetes, was induced by berberine. MRAK052686 co-localize at the 3′UTR of Zbtb20, coding for a protein regulating glucose homeostasis. The co-expression of MRAK052686 and Zbtb20 increases the level of the protein, improving glucose homeostasis [62].

## 8. Nanotechnological Strategies to Improve the Delivery of Berberine

**Figure 4:**
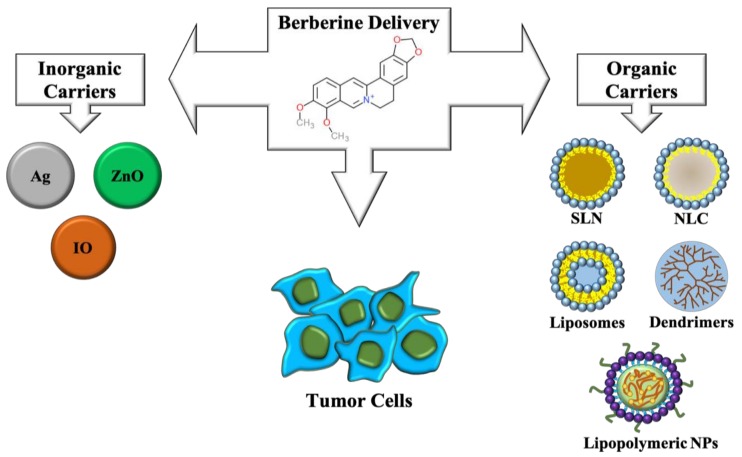
Berberine delivery strategies. On the left, inorganic nanocarriers are shown: Ag, Silver nanoparticles; ZnO, zinc oxide nanoparticles; IO, Iron oxide nanoparticles. On the right, organic nanocarriers are shown: SLN, solid lipid nanoparticles; NLC, nanostructured lipid carriers; Liposomes; Dendrimers; Lipopolymeric nanoparticles.

Due to its outstanding antitumoral properties, many efforts have been devoted in designing carriers for berberine delivery as an anti-cancer therapeutic agent. Both inorganic and organic nanomaterials have been exploited for this purpose (shown in Figure 4).

Silver nanoparticles proved successful in delivering berberine to human tongue squamous carcinoma SCC-25 cells, blocking cell cycle and increasing Bax/Bcl-2 ratio gene expression, thus indicating activation of pro-apoptotic pathways triggered by mitochondrial dysfunction [78]. Interestingly, silver nanoparticles carrying berberine displayed elevated cytotoxicity in different breast cancer cell lines and inhibition of tumor growth in vivo [79]. The same research group fabricated citrate-capped silver nanoparticles loaded with berberine and conjugated to polyethylene glycol-functionalized folic acid and demonstrated that they were able to induce apoptosis and variations in gene expression in breast cancer cells. In MDA-MB-231 athymic nude mice models, a significative reduction of tumor progression was observed [80].

Zinc oxide nanoparticles carrying berberine were recently synthesized for lung cancer therapy. They displayed antiproliferative activity in A549 cells and no significant toxicity in vivo. Moreover, due to the intrinsic properties of ZnO, these materials have been exploited as photothermal therapy (PTT) agents, leading to thermal ablation of cancer cells [81].

An innovative approach has been recently adopted by creating iron oxide nanoparticles complexed with hypoxic cell sensitizer sanazole together with berberine, able to target hypoxic tumors in vivo. Hypoxia is known to induce expression of HIF-1-alpha and consequent activation of angiogenesis related genes. Interestingly, after administration of nanoparticles, transcriptional downregulation of these and many genes linked to cell proliferation and metastasis was observed and clearly correlated with a reduction of tumor volume in vivo [82].

Many studies have demonstrated the feasibility of designing organic nanoparticles for berberine delivery. Solid lipid nanoparticles have been synthesized with good stability, high berberine loading and huge entrapment efficiency, essential parameters for successful clinical evaluation. These nanomaterials inhibited cell proliferation of MCF-7, HepG2, and A549 cancer cells inhibited cell cycle progression and apoptosis in MCF-7 cells [83].

Nanostructured lipid carriers efficiently delivered berberine to H22 hepatocarcinoma cells with high antitumor efficacy [84]. Lipid nanoparticles covered with the self-tumor targeting polymers lactoferrin and hyaluronic acid were fabricated for berberine and rapamycin delivery to lung cancer cells and displayed improved internalization and selective cytotoxicity. Detectable reduction in the number of lung foci and vascular endothelial growth factor levels were further observed [85]. The same approach was adopted by administering inhalable nanoparticles to in vivo models of lung cancer, achieving a significant decrease in lung weight, reduction in lung adenomatous foci number and diameter and in angiogenic markers expression [86].

Amine terminated G4 PAMAM have been developed with conjugated berberine and demonstrated specific cytotoxicity in different human breast cancer cell lines [87]. Lipopolymeric micelles have been developed that greatly improved berberine water solubility up to 300% with low toxicity and induced apoptosis in treated monolayer and spheroid cultures of human prostate carcinomas [88]. Finally, berberine loaded folate acid modified chitosan nanoparticles demonstrated effective in inhibiting proliferation and migration and inducing apoptosis and necrosis in human nasopharyngeal carcinoma cells CNE-1 [89].

## 9. Conclusions

Berberine has emerged as an excellent natural product having significant biological activity [90]. It has demonstrated premium activity against different cancers. Increasingly sophisticated cutting-edge research has uncovered tremendous chemopreventive ability of berberine to modulate signaling pathways [91,92].

In the last few years, many studies have been focused on unraveling intrinsic properties of berberine and its ability to interfere with intracellular pathways. In particular, there is an increasing interest in exploiting this natural compound as an effective anticancer drug. Although some issues remain to be solved, such as its poor water solubility/stability and low bioavailability, many nanotechnological approaches have allowed the design of ad hoc delivery systems, making berberine application for cancer treatment feasible. Many different nanocarriers, based both on inorganic and organic materials, have been developed and proven to be effective in overcoming some of the above-mentioned issues and in delivering berberine in an extremely efficient manner in many different cancer experimental models. In the near future, further research will provide crucial answers needed to pave the way to berberine clinical application.

## Abbreviations

Breast cancer cells	MDA-MB-231, MCF-7, MDA-MB-468
hepatocellular carcinoma cells	SNU-182, HepG2, Hep3B, H22 SMMC-7721
gastric cancer cells	SGC7901, AGS
lung carcinoma	A549
myeloma	U266 cell line
ovarian cancer cell line	SKOV3
Akt	protein kinase B
AMPK	AMP-activated protein kinase
AQP-1	aquaporin 1
APC	adenomatous polyposis coli
Bcl-2	B-cell lymphoma 2
CCND1	cyclin D1
c-FLIP	cellular FADD like interdeukin-1-p-converting enzyme inhibitory protein
CK1	casein kinase 1
COX2	cyclooxygenase-2
CREB	cAMP response element-binding protein
DISC	death inducing signaling complex
DNMT1	DNA (cytosine-5)-methyltransferase 1
DR	death receptor
EGFR	epidermal growth factor receptor
ERK	extracellular signal-regulated kinases
EVs	extracellular vesicles
GADD45α	growth arrest and DNA damage 45α
GLUT1	glucose transporter 1
HIF-1α	hypoxia-inducible factor 1α
HK2	hexokinase 2
HUVECs	human umbilical vein endothelial cells
IO	iron oxide
JAK-STAT	Janus kinases-signal transducer and activator of transcription proteins
LDHA	lactate dehydrogenase A
lncRNAs	long noncoding RNA
LRP	low-density lipoprotein receptor-related protein
MAPK	mitogen-activated protein kinases
MARCKS	myristoylated alanine-rich C-kinase substrate
MMP	matrix metalloproteinases
mTOR	mammalian target of rapamycin
NF-κB	nuclear factor kappa-light-chain-enhancer of activated B cells
NLC	nanostructured lipid carriers
NP	nanoparticles
NPC	nasopharyngeal carcinoma
PI3K	phosphoinositide 3-kinases
RXRα	retinoid X receptor α
SIRT1	sirtuin 1
SLN	solid lipid nanoparticles
T2D	type 2 diabetes
TAFs	tumor-associated fibroblasts
TARID	TCF21 antisense RNA inducing demethylation
TNF	tumor necrosis factor
TRAIL	TNF-related apoptosis-inducing ligand
ULK1	Unc-51 like autophagy activating kinase
VEGF	vascular endothelial growth factor
ZnO	zinc oxide.
